# The role of the multidisciplinary tumor board (MDTB) in the assessment of pancreatic cancer diagnosis and resectability: A tertiary referral center experience

**DOI:** 10.3389/fsurg.2023.1119557

**Published:** 2023-02-17

**Authors:** Giuseppe Quero, Davide De Sio, Claudio Fiorillo, Roberta Menghi, Fausto Rosa, Giuseppe Massimiani, Vito Laterza, Chiara Lucinato, Federica Galiandro, Valerio Papa, Lisa Salvatore, Maria Bensi, Antonio Pio Tortorelli, Vincenzo Tondolo, Sergio Alfieri

**Affiliations:** ^1^ Pancreatic Surgery Unit, Fondazione Policlinico Universitario “Agostino Gemelli” IRCCS, Largo Agostino Gemelli, Rome, Italy; ^2^ Gemelli Pancreatic Advanced Research Center (CRMPG), Università Cattolica del Sacro Cuore di Roma, Rome, Italy; ^3^ Comprehensive Cancer Center, Fondazione Policlinico Universitario Agostino Gemelli, IRCCS, Rome, Italy; ^4^ General Surgery Unit, Fatebenefratelli Isola Tiberina – Gemelli Isola, Via di Ponte Quattro Capi, Roma, Italy

**Keywords:** pancreatic cancer, multidisciplinary, resectability, diagnosis, treatment response

## Abstract

**Background:**

The introduction of multidisciplinary tumor boards (MDTBs) for the diagnostic and therapeutic pathway of several oncological disease significantly ameliorated patients' outcomes. However, only few evidences are currently present on the potential impact of the MDTB on pancreatic cancer (PC) management. Aim of this study is to report how MDTB may influence PC diagnosis and treatment, with particular focus on PC resectability assessment and the correspondence between MDTB definition of resectability and intraoperative findings.

**Methods:**

All patients with a proven or suspected diagnosis of PC discussed at the MDTB between 2018 and 2020 were included in the study. An evaluation of diagnosis, tumor response to oncological/radiation therapy and resectability before and after the MDTB was conducted. Moreover, a comparison between the MDTB resectability assessment and the intraoperative findings was performed.

**Results:**

A total of 487 cases were included in the analysis: 228 (46.8%) for diagnosis evaluation, 75 (15.4%) for tumor response assessment after/during medical treatment, 184 (37.8%) for PC resectability assessment. As a whole, MDTB led to a change in treatment management in 89 cases (18.3%): 31/228 (13.6%) in the diagnosis group, 13/75 (17.3%) in the assessment of treatment response cohort and 45/184 (24.4%) in the PC resectability evaluation group. As a whole, 129 patients were given indication to surgery. Surgical resection was accomplished in 121 patients (93.7%), with a concordance rate of resectability between MDTB discussion and intraoperative findings of 91.5%. Concordance rate was 99% for resectable lesions and 64.3% for borderline PCs.

**Conclusions:**

MDTB discussion consistently influences PC management, with significant variations in terms of diagnosis, tumor response assessment and resectability. In this last regard, MDTB discussion plays a key role, as demonstrated by the high concordance rate between MDTB resectability definition and intraoperative findings.

## Introduction

The progressive treatment centralization in high-volume centers of the majority of surgical diseases has increasingly gained relevance in the last decade (1). For instance, multiple evidences demonstrated significant advantages in the diagnostic and therapeutic pathways of oncological diseases, such as breast, thoracic, gynecologic, urologic, hepatic and gastrointestinal malignancies, when treated in specialized centers (2–7). Such centralization is based on the multidisciplinary approach to the disease of interest, through the recent institution of multidisciplinary tumor boards (MDTBs). MDTBs involve multiple professional figures such as surgeons, radiologists, medical and radiation oncologists, endoscopists and pathologists, with the aim of guarantying an appropriate and tailored disease care, ensuring, at the same time, an adequate use of healthcare resources. This inevitably brought to the recommendation of the multidisciplinary coordination of care of several diseases by national and international guidelines, including also the National Comprehensive Cancer Network (NCCN) (8).

In this context, given the high complexity of pancreatic cancer (PC) management, the MDTB could potentially give a significant support for an appropriate decision-making. For instance, the incidence rate of PC is progressively increasing. It currently represents the fourth cause of cancer-related death, and it is expected to become the second by 2030 (9, 10). Nowadays, surgery still represents the gold standard of treatment. However, according to recent data, less than 20% of patients affected by PC may be eligible for surgery with curative intent, while the majority of them frequently presents with a locally advanced or metastatic disease at first diagnosis (11). It is, thus, implicit how the MDTB could be of paramount importance for an appropriate diagnosis and resectability assessment of PC. Indeed, the determination of pancreatic tumor resectability (especially for borderline and locally advanced tumors) is the most challenging step in PC management. It is based on the evaluation of tumor involvement of major vessels and on the degree assessment of the contact with them (12). This makes resectability evaluation a prerogative of specialized radiologists in association with several other professional figures in order to guarantee an adequate patient selection for surgery.

Despite these premises, only few evidences are currently present on the potential impact of MDTB on PC management (13–15), while no study specifically evaluated the role of MDTB on PC resectability, especially in terms of concordance between first assessment and MDTB evaluation and between the MDTB evaluation and intraoperative findings. Aim of this study is, thus, to evaluate how the MDTB may have influenced the surgical decision-making and how often the consensus decision of resectability was validated by the intra-operative findings.

## Materials and methods

### Study population

After Institution Review Board approval, all patients with a proven or suspected diagnosis of PC referred to the MDTB of the Fondazione Policlinico Universitario Agostino Gemelli IRCCS of Rome from October 2018 to March 2020 were retrospectively enrolled in the study. Patients affected by pancreatic cysts were also included as well as cases of pancreatitis with suspected underlying tumors. This last subgroup included patients with a recent episode of acute pancreatitis as well as those affected by chronic pancreatitis in follow up at the dedicated outpatient center for pancreatic diseases. The following patients' demographic and clinical data were recorded: age, sex, medical history, symptoms and signs related to the pancreatic disease, laboratory test results and histopathological findings (when available), at the time of referral. Radiological exams available for revision during the meetings included: computed tomography (CT) and/or magnetic resonance imaging (MRI) and/or positron emission tomography (PET) and/or endoscopic ultrasonography (EUS).

### PC-MDTB

At our institution, the PC-MDTB takes place once a week and involves surgeons, radiologists, gastroenterologists, clinical and radiation oncologists, endoscopists and pathologists. Proposal for discussion is at discretion of the attending physician, determining a certain heterogeneity of stages in the diagnostic-therapeutic pathway in which patients are discussed. The agenda with all the cases proposed for discussion is created the day before the meeting to allow the radiologists to upload and preview the radiological images. During the MDTB, available medical records and radiological exams are routinely reviewed case-by-case and, after discussion among the different members, a consensus recommendation is produced. Whenever diagnostic data are considered insufficient, additional exams such as radiological exams and/or endoscopic procedures with or without biopsy are prescribed and programmed using dedicated slots committed to patients discussed at the MDTB.

Tumor resectability is assessed by the experts attending the meeting, defining PCs as resectable, borderline resectable, locally advanced and metastatic in accordance with the current treatment guidelines, including the National Comprehensive Cancer Network (NCCN) guidelines (8). The Italian Medical Oncology Association (16) and ESMO (17) guidelines are followed to propose a neoadjuvant, adjuvant or palliative treatment.

Pancreatic cystic tumors with a potential indication to surgical resection such as IPMN and mucinous cystadenomas, are also discussed and the final recommendation is given according to the current International Consensus Guidelines 2016 (18).

Pre-MDTB diagnosis and staging, defined by the physician who presents the case, are prospectively collected as well as the subsequent post-MDTB diagnosis, staging and recommendation, indicated by the multidisciplinary decision after the meeting.

For the study purposes, discrepancies between the pre- and post-MDTB decisions were recorded. When surgical resection was proposed, intra-operative findings were then compared to the post-MDTB diagnosis and staging. For this last analysis, only patients with a diagnosis of pancreatic adenocarcinoma (PDAC) after MDTB discussion were included.

### Study outcomes

The primary outcome of the study was to evaluate the changes in PC diagnosis and management after MDTB discussion. To accomplish this purpose, clinical cases were classified according to the request for first discussion into: PC diagnosis, PC response to oncological and/or radiation treatment and PC resectability assessment. Secondary outcome was to compare the assessment of tumor resectability at the multidisciplinary discussion with the intra-operative findings during surgical exploration.

### Statistical analysis

Categorical variables are presented as numbers and percentages, and continuous variables are presented as median and range (min-max). All data were analyzed by SPSS v25® (IBM, Chicago, IL).

## Results

From October 2018 to March 2020, 487 clinical cases were discussed at the PC-MDTB of the Fondazione Policlinico Universitario Agostino Gemelli IRCCS of Rome. Of them, 101 (28.7%) needed rediscussion: 76 cases were discussed twice, 15 cases three times and 10 patients four times. The most common reasons for rediscussion were the need for additional examinations after the first evaluation and revaluation during chemotherapy.

Clinico-demographic characteristics of the study population are reported in Table 1. Median age at the time of presentation was 67 (25–91) years. Two-hundred and forty-seven patients (50.7%) were male and 240 female (49.3%). Pancreatic adenocarcinomas represented the most frequent disease proposed for discussion (307%–63%), followed by pancreatitis (84%–17.3%), IPMNs (39%–8%) and cystic lesions (57%–11.7%).

**Table 1:** Clinico-demographic characteristics of the study cohort.

	N. of clinical cases (*n* = 487)
**Rediscussion, *n* (%)**	101 (28.7)
**Age (years), median (range)**	67 (25–91)
**Sex, *n* (%)**	
*Male*	247 (50.7)
*Female*	240 (49.3)
**Pancreatic disease (pre-MDTB), *n* (%)**	
*Adenocarcinoma*	307 (63)
*Pancreatitis*	84 (17.3)
*IPMN*	39 (8)
*Cystic lesions*	57 (11.7)

Of the study population, 228 (46.8%) patients were discussed for diagnosis evaluation, 75 (15.4%) patients for tumor response assessment after/during chemotherapy and/or radiotherapy and 184 (37.8%) patients for PC resectability. As a whole, MDTB discussion led to a change in 89 out of 487 patients (18.3%).

Regarding diagnosis evaluation, PC-MDTB brought to a change rate of 13.6% (31 patients out of 228). Specifically, 6 lesions out of 31 (19.3%) firstly diagnosed as benign cysts resulted to be IPMN in 4 cases and pancreatitis in the remaining 2 patients. Similarly, 7 out of 31 (22.6%) lesions, firstly presented as IPMN, were diagnosed as benign pancreatic cyst in 3 cases and pancreatitis in 4 cases after multidisciplinary discussion. Four patients (12.9%) with an initial diagnosis of pancreatitis resulted to be affected by IPMN (2 cases) and a resectable pancreatic tumor (2 cases). The remaining 14 patients (45.1%) accessed to the PC-MDTB with a diagnosis of PDAC. After discussion, 2 (6.4%) were diagnosed as IPMNs, 2 (6.4%) as benign cystic lesions and 10 (32.2%) as pancreatitis. As a whole, of the group of patients presented for a diagnostic assessment, 43 (18.9%) underwent surgery for a resectable PDAC (36 patients), a mucinous cystoadenoma in 4 cases and IPMN in 3 cases.

Of the 75 cases presented for the assessment of tumor response after/during chemotherapy and/or radiotherapy, change in terms of restaging was evidenced in 13 patients (17.3%). Indeed, 4 lesions out of 37 judged (18.8%) as stable disease before discussion showed a progression disease, instead. Among the 26 patients presented with a partial response, 5 (19.2%) were considered to have a stable disease. Moreover, of the 2 patients presented with a complete response, 1 (50%) was considered as having a partial response and 1 (50%) a stable disease. Finally, 2 patients out of 10 (20%) presented with a disease progression were classified as stable disease after MDTB discussion. Thus, 8 out 75 patients (10.6%) were given indication to surgery for resectable disease (4 cases) and for a borderline lesion (4 cases).

**Figure 1:**
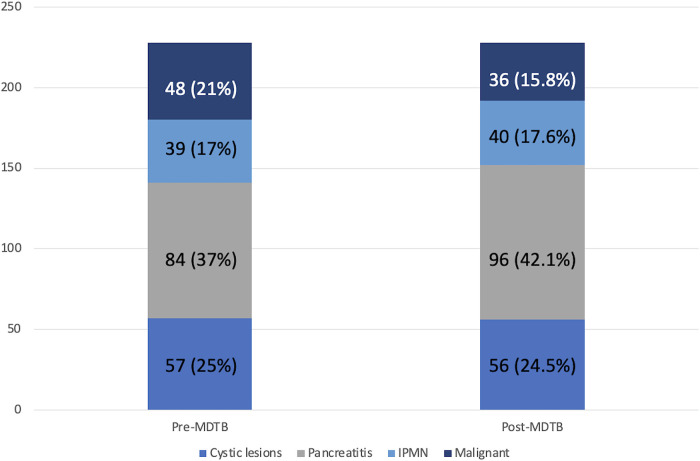
Pc diagnosis variation after MDTB discussion.

**Figure 2:**
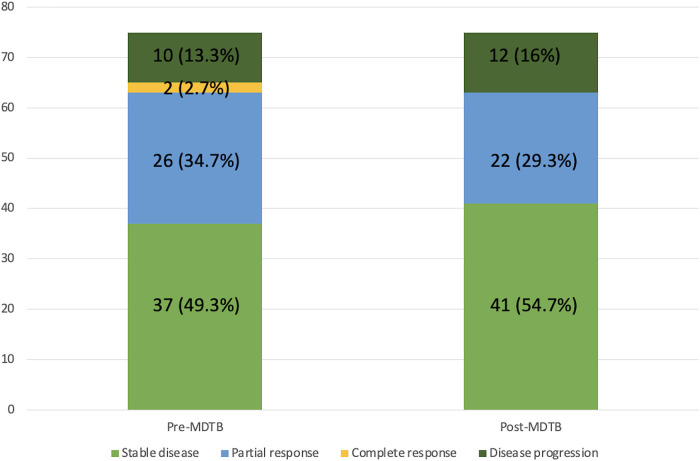
Tumor response assessment variation after MDTB discussion.

Diagnostic changes and tumor response assessment in accordance to the PC-MDTB discussion is reported in Figures 1, 2.

### MDTB assessment of Pc resectability (Figure 3)

One-hundred and eighty-four patients out of 487 (37.8%) affected by PDAC were presented to assess resectability at first discussion. As a whole, 77 (41.8%) were classified as resectable, 43 (23.4%) as borderline, 36 (19.6%) as locally advanced and 28 (15.2%) with a suspicion of metastatic disease. After the multidisciplinary discussion, resectability assessment changed in 45 cases (24.4%). Specifically, of the 77 firstly classified resectable PCs, 6 (7.8%) were defined as borderline and 6 (7.8%) as locally advanced. Similarly, after MDTB discussion, of 43 borderline lesions, 2 (4.7%) were judged resectable, 15 (34.9%) locally advanced and 2 (4.7%) metastatic. Of note, of 36 lesions presented as locally advanced, 1 (2.8%) was categorized as resectable, 1 (2.8%) as borderline and 7 (19.4%) as metastatic. All 28 patients with a suspicion of metastatic disease were confirmed as metastatic. Thus, based on the MDTB discussion, 99 out of 184 (53.8%) patients were given indication to chemotherapy. As a whole, MDTB changed the disease management in 35 (19%) cases.

**Figure 3:**
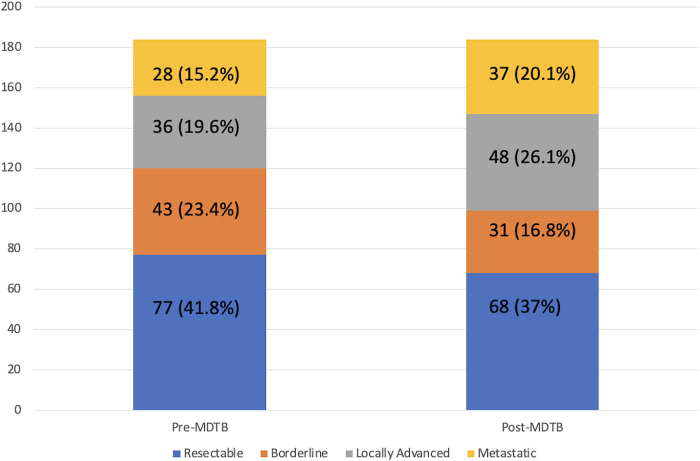
Pc resectability change after MDTB discussion.

### Correspondence between MDTB resectability assessment and intra-operative findings

As a whole, 129 patients were given indication to surgery after MDTB discussion: 101 of them (78.3%) were classified as resectable and 28 (21.7%) were defined as borderline. Surgical resection was accomplished in 121 patients (93.7%). In terms of correspondence between MDTB resectability assessment and intraoperative finding, 100 out 101 patients (99%) were confirmed as resectable, while 1 patient (1%) presented a borderline lesion requiring a tangential resection of the superior mesenteric vein. Of the 28 patients firstly classified as borderline, 2 (7.1%) presented a resectable tumor, 18 (64.3%) were confirmed as borderline, while the remaining 8 patients (28.6%) had a contraindication to resection for liver metastases in 2 cases and circumferential infiltration of the superior mesenteric artery in 6 cases. Of the 18 bordeline lesions, 16 required a venous tangential resection while in 2 cases a resection of the superior mesenteric vein with a primary anastomosis was needed. Thus, MDTB discussion successfully assessed resectablity in 91.5% of cases (118 out of 129 patients).

**Figure 4:**
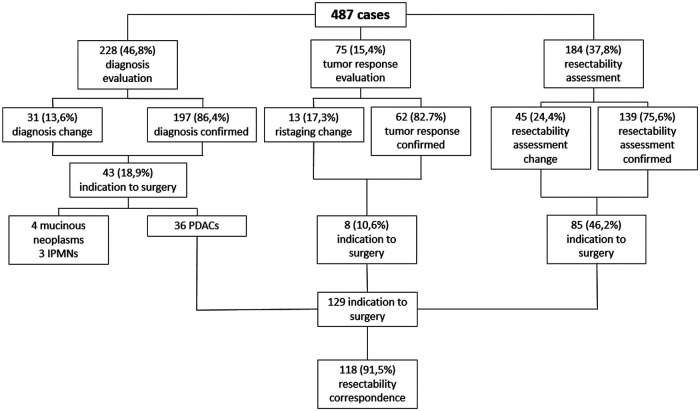
Diagram of the MDTB management and outcomes.

An explicative diagram of the MDTB outcomes has been reported in Figure 4.

## Discussion

The recent implementation of the treatment centralization of the majority of oncological diseases, with the consequent introduction of dedicated MDTBs, has brought significant advantages in terms of short- and long-term outcomes. Specifically, multiple authors demonstrated a more accurate tumor staging, a lower incidence of post-operative complications as well as a shorter time interval between diagnosis and treatment (7, 13, 19, 20). In addition, better prognosis has been reported for the majority of oncological diseases when cases are discussed at dedicated MDTBs (21).

Despite these premises, only few evidences are currently present on the potential impact of MDTB in the diagnostic and therapeutic management of PC (13–15). Moreover, no data are currently present in the literature on the correspondence between the MDTB definition of resectability and the intraoperative findings. For these reasons, with the aim to give our contribution to this relevant topic, we performed a retrospective analysis on the cases of pancreatic diseases evaluated at the PC-MDTB in a time-lapse of almost two years. Data and outcomes were consequently evaluated according to the clinical need presented, namely diagnosis evaluation, tumor response to medical treatment and resectability assessment. According to our data, MDTB discussion led to a diagnostic change in the 13.6% of cases. Similarly, tumor response to chemotherapy was changed by the multidisciplinary discussion in the 17.3% of patients. Of note, PC resectability changed in the 24.4% of cases with a concordance rate between MDTB classification and intraoperative findings of 91.5%. According to these data, it is thus implicit the potential advantage deriving form a multidisciplinary approach to the disease.

Regarding the potential impact of the MDTB on PC diagnosis, discrepancy rate was evidenced in 31 out of 228 cases (13.6%). This data further underline the relevance of imaging revision by dedicated radiologists with extensive experience in pancreatic disease. In this regard, it is common knowledge how challenging may be to perform a correct diagnosis, especially in case of small size tumors and for those lesions not significantly deforming the pancreatic parenchyma (22). Indeed, multiple evidences have already demonstrated a higher diagnostic accuracy and a more appropriate disease staging when radiological images are revised by expert radiologists (14, 23). Such a high rate of discrepancy may, thus, be justified by the execution of radiological examinations in low volume centers, and by the lack of specific and dedicated diagnostic pathways with the possibility of performing second-level exams, causing misinterpretation of the disease diagnosis.

A significant discrepancy rate between the MDTB case presentation and the final result of the discussion was also evidenced for the evaluation of the tumor response to chemo- and/or radiotherapy. We specifically evidenced a discrepancy rate of 17.3% with a consequent rate of change in the disease management of 10.6%. This inevitably implied the modification of the treatment strategy reserved to these patients. Even in this case, since revaluation after treatment is widely recognized as insidious and extremely challenging, the role of the radiologist is still fundamental for an appropriate re-classification of the disease (24). Moreover, the multidisciplinary discussion involving specialized physicians play a key role in setting the patient in an appropriate clinical context. This inevitably leads to a maximization of the treatment strategy and in the amelioration of patients' outcome affected by PC.

According to our data, tumor resectability assessment presented the highest discordance rate (45 out of 184 cases, 24.4%). This value is extremely worrying, especially for the relevance that an adequate disease staging represents for patients' prognosis. In this regard, some author already reported an underuse of pancreatic resection for small and resectable lesions in a nationwide cohort of patients, with the highest tendency towards non-treatment strategies especially in low volume centers (25). This further underlines the importance of a multidisciplinary approach for an appropriate disease staging and resectability assessment, involving contemporarily specialized figures such as radiologists, surgeons and clinicians. This is of paramount importance especially in the discrimination between locally advanced and borderline lesions, due to the presence of no uniform guidelines and several different classification (26). The concomitance of multiple figures is thus fundamental not only for the adequate radiological assessment but also for the risk/benefit ratio evaluation, with the aim of guarantying a patient-tailored treatment. Although current evidences have already demonstrated the value of PC-MDTB in the appropriate classification of PCs as resectable, borderline and locally advanced (13–15), no author demonstrated the effective correspondence of the MDTB assessment and the intra-operative findings. Such a comparison is fundamental in order to further confirm the beneficial role of MDTB for an adequate patient selection for surgery. In this regard, we observed a concordance rate of 91.5%, with the highest value reached in the definition of resectable lesion (99%). On the contrary, although with a consistent concordance rate (64.3%), borderline lesions have been confirmed as the most challenging to be appropriately assessed. This further supports the major difficulty in the correct CT images interpretation especially when conducted in low-volume centers. Indeed, failure in the accurate radiological assessment of borderline lesions may potentially lead to unnecessary laparotomies. Although the extent of this problem is still unknown, Katz et al. evidenced a 24% rate of nontherapeutic laparotomies for an inadequate staging of borderline PCs (27). Based on these data, it is likely that such percentage may be even higher in case of surgical treatment in non-specialized centers without a multidisciplinary approach to the pancreatic disease.

Although our data demonstrate how relevant is the multidisciplinary approach to PC, it is undeniable the need for further amelioration in the diagnosis and treatment of PC. In this context, the introduction of new technologies in medicine is gradually offering chances to improve patients' outcomes. In particular, a significant contribution is given by artificial intelligence (AI), conceived to support physician for the most appropriate decision making. This could be particularly valuable for the treatment of challenging diseases such PC. Despite still in its infancy, AI applications to PC have demonstrated promising results from diagnosis to treatment (28–30). Based on these preliminary data, we do believe that such a new technology may give a relevant support to clinicians especially in the context of a multidisciplinary approach, where all specialized medical figures may be supported by AI technology in order to guarantee the most adequate diagnostic-therapeutic pathway.

Our study presents some limitations. Although we confirmed the relevant role of the MDTB in the diagnostic and therapeutic pathway of PCs, the absence of a comparative no-MDTB group does not permit to draw solid conclusions on the potential benefits in terms of short-term outcomes and potential advantages in terms of time-elapse between diagnosis and treatment. Moreover, it would have been of great interest to have a long-term prognostic evaluation of the study cohort, in order to assess potential advantages in terms of prognosis.

In conclusion, we confirmed the fundamental role of MDTB in PC diagnosis, tumor response evaluation and resectability assessment as demonstrated by the significant change in the treatment strategy we detected. Moreover, for the first time in the literature, the relevance of PC-MDTB has been furthermore supported by the high concordance rate evidenced between the post-discussion resectability evaluation and the intra-operative findings. It is, however, undeniable the need for further studies to confirm our data, comparing the clinical results of the MDTB discussion to previous retrospective cohorts that did not benefit from a multidisciplinary assessment.

## Data Availability

The raw data supporting the conclusions of this article will be made available by the authors, without undue reservation.
